# A Sanitary Organization of Women

**Published:** 1890-07

**Authors:** 


A Sanitary Organization of Women.—The women of Brooklyn have united to form a Ladies' Health Protective Association, similar to one which has for years been so useful in New York City. The lines of its work will be in the direction of such nuisances as offensive pursuits, uncared for tenement houses, and filthy streets. The wives of physicians form a considerable proportion of the organization.—*Weekly Medical Review.*

